# Effect of Rotary Swaging on Mechanical Behaviors of Axle Steel Rod

**DOI:** 10.3390/ma17112525

**Published:** 2024-05-24

**Authors:** Tiantai Tian, Hongtu Xu, Huaibei Zheng, Wenbin Zhan, Yu Zhang, Haosong Zhu, Qi Zhang

**Affiliations:** 1 State Key Laboratory of Metal Material for Marine Equipment and Application, Ansteel Group Corporation, Anshan 114009, China; tiantai319@stu.xjtu.edu.cn (T.T.);; 2 School of Mechanical Engineering, Xi’an Jiaotong University, Xi’an 710049, China

**Keywords:** axle steel, fatigue, fracture, mechanical properties, rotary swaging

## Abstract

The short-chain forming process using rotary swaging (RS) is an important method of achieving the manufacturing of lightweight axles. Axle steel, like 42CrMo, is widely used in many types of axles and shafts; however, there is no existing research on rotary-swaged axle steel’s mechanical properties. It makes sense to carry out a comprehensive study on the effect of RS on the mechanical behaviors of axle steel rods. In this study, a 42CrMo steel rod was processed by RS through ten passes. The tensile properties, torsion properties, compression properties, and fatigue properties were tested. There was an overall improvement in the torsional and fatigue performance after RS. Combined with a finite element analysis (FEM), the uneven distribution of the dislocations and existence of the elongation material were inferred to have caused the different modes of the mechanical behaviors. Fracture surfaces were analyzed and the results showed that the fracture pattern had changed. There existed a competitive relation between the internal fatigue cracks and external cracks, which could be attributed to uneven strain hardening. This research proved the advantages of RS in the processing of axle parts, which mainly benefitted the torsional working conditions, and provided evidence for a new processing route for lightweight axles with RS.

## 1. Introduction

With the development of the low-carbon industry, lower energy consumption and more efficient manufacturing are becoming increasingly important. For the automobile industry, lightweight design as well as short-chain forming are popular ways to produce less carbon emissions. Shaft parts occupy a key role in automobile transmission systems, and one of the best ways to reduce the weight of these components will be to design and manufacture them as hollow components [1]. Forming processes, especially those based on short-chain lightweight design of shaft parts, are an effective solution to reduce the vehicle weight, thus saving energy in the vehicle’s long service life cycle [2].

**Figure 1:**
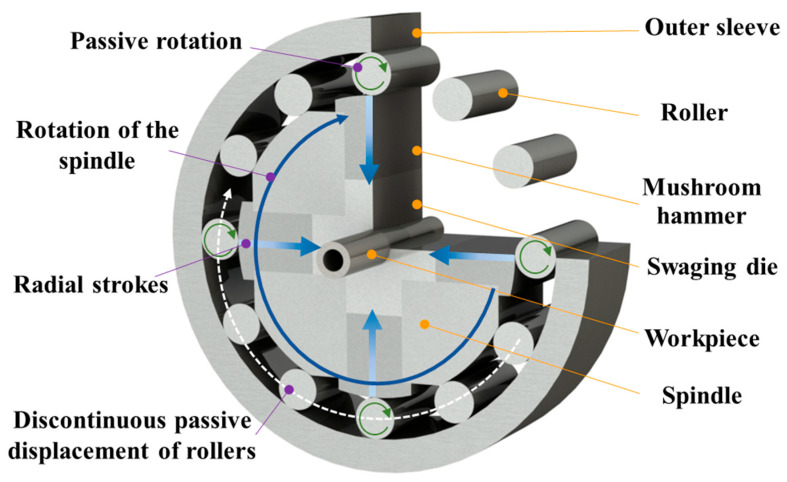
Schematic diagram of RS.

Rotary swaging (RS) is an incremental forming process that can be used to produce hollow shafts by reducing the diameter of a workpiece in small steps through the oscillating action of the dies [3,4]. The forming principle is illustrated in Figure 1: The spindle drives the mushroom hammers and swaging dies to rotate, causing them to move outwards due to the centrifugal force. When the mushroom hammers pass the roller, they are forced to push the dies into the closed condition. Meanwhile, rollers will also display passive rotation. The RS process has the advantages of work hardening, close tolerances, and smooth surfaces [5]. This technology enables partial increases in the wall thickness of hollow shafts within a very short process time and can be used to shape the parts externally and internally. Using this process, tolerances of up to IT8 or 9 can be reached. Thus, the finishing process can be simplified significantly or partially neglected, achieving short-chain processes [6].

**Figure 2:**
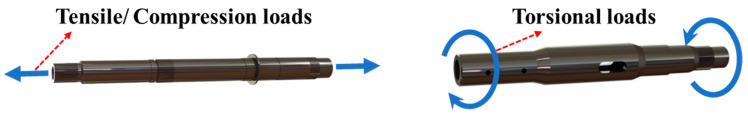
Working conditions of different axles.

It is well known that 42CrMo steel has the good properties of high strength, ductility, toughness and wear resistance, and has been widely applied in critical shafts and axles with large cross-sections, complex shapes, and heavy workloads [7,8]. These axles or shafts are basically used in high-speed railways and vehicles. RS technology is a very promising method to achieve high-performance forming. As Figure 2 shows, in different application scenarios, original billets are processed as different kinds of shaft parts, which can bear different working loads [9]. The strengthening process, which modifies the material, will have a large influence on the properties of the shafts [10]. In this respect, it is extremely necessary to study the mechanical properties of 42CrMo steel processed by RS.

The impact of the infeed RS of steel bars on the material flow and plastic deformation has been investigated by Moumi and Liu et al. through FEM [11,12,13,14]. In terms of the material properties that arise after RS, many scholars have conducted extensive research on different materials.

Charni et al. studied the tensile properties and three-point bending fatigue properties of S355 steel components processed by RS and found that the yield strength had doubled and the bending fatigue strength had increased by 6.47~12.5% [15]. Ahmed et al. found that the rotational bending fatigue strength of swaged 316 L stainless steel increased by 40% [16]. The study by de Salvo et al. indicated that the fatigue strength of swaged Al wire was increased by 20%, and the dispersion of the fatigue test data was also reduced [17]. Abdulstaar et al. found that the fatigue limit of the swaged fine-grained 1050 Al was about 125% higher than that of the original coarse-grained material [18]. They also pointed out that the more limited ductility and hardening ability of the swaged material increased the fatigue crack growth rate [19]. Klumpp et al. found a strengthening effect of cold RS on the uniaxial mechanical behavior of Inconel 718 superalloy. They also found that the fatigue crack growth rate was significantly increased [20,21]. Other research investigated the tensile properties of pure copper [22], titanium alloy [23], magnesium alloy [24], and tungsten heavy alloy [25].

RS and radial forging are commonly used in industry for manufacturing gun barrels. Therefore, steel materials similar to 42CrMo, such as 30SiMn2MoVA steel, have also been extensively investigated. Fan et al. studied the axial and circumferential mechanical properties of radial forged gun barrels made of 30SiMn2MoVA steel and explored the influence of annealing processes on both mechanical properties [26]. They also investigated the effect of the amount of deformation on the plastic anisotropy [27,28]. Although there are few studies on the RS of 42CrMo shaft components, manufacturing processes based on plastic forming such as wedge cross-rolling have been widely researched. Cui et al. studied the axial-infeed incremental rolling process of 42CrMo spline shafts and improved the hardness [29]. Szala et al. conducted research on the cold forging manufacturing of hollow shafts with flanges made of 42CrMo and obtained the hardness distribution [30]. Pater et al. [31] and Shu et al. [32] have been engaged in rolling process research on 42CrMo axles and achieved significant reductions in diameter; however, they have not conducted sufficient research on the mechanical properties.

In summary, the RS process has good application prospects for the short-chain forming of axles or shafts, so the mechanical properties arising after RS are of great significance. Currently, there has not been a comprehensive assessment of the mechanical properties of 42CrMo steel after cold RS. Carrying out the research on the performance of rotary-swaged 42CrMo will provide an extremely valuable reference for the future applications of RS technology on various axles.

## 2. Materials and Methods

### 2.1. Materials

The 42CrMo axle steel used in the present study was produced by Henan Jiyuan Iron & Steel Co., Ltd, Jiyuan, China and the chemical composition is presented in Table 1. No additional heat treatment was conducted on the material.

The rod steel was cut at dimensions of Φ25 mm × 1500 mm. One batch of the received rod steel was kept in its as-received state, and the diameter of the other batch was then reduced from Φ25 mm to Φ15 mm by RS. Because of the high strength of the materials, the RS process was completed in ten passes; thus, the rods were incrementally swaged to their final dimensions.

By defining the swaging ratio as $\varphi =\left|ln\left(\frac{d_{1}^{2}}{d_{0}^{2}}\right)\right|$, in which *d*_0_ represents the initial diameter and *d*_1_ represents the swaged diameter, the final swaging ratio is calculated as φ = 1.022.

The RS process was carried out on an X50 RS machine produced by Xi’an Innovation Precision Instrument Co., Ltd., Xi’an, China. The microstructure investigations were carried out on an optical OLYMPUS BX3M microscope (Olympus, Waltham, MA, USA). Specimens of both material batches were prepared and finally polished (diamond spray compounds with 0.25 μm size) for microstructure observation. A 4% nital solution was used to investigate the microstructure’s evolution.

### 2.2. Mechanical and Fatigue Testing

**Figure 3:**
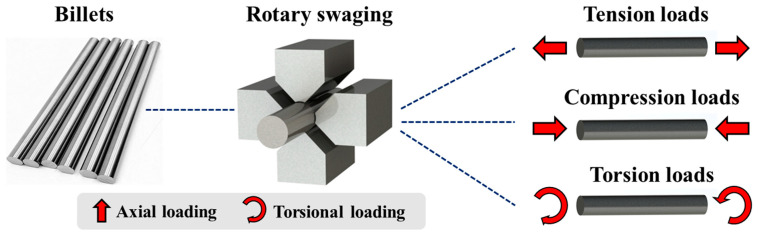
Different mechanical conditions of rotary-swaged rods.

Considering different working conditions of axles, the swaged rods were tested under different conditions, as shown in Figure 3.

**Figure 4:**
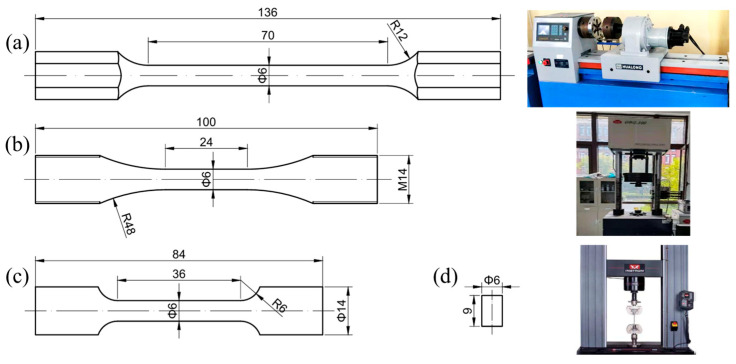
Specimen design and testing machines: (**a**) torsion specimen; (**b**) fatigue specimen; (**c**) tensile specimen; (**d**) compression specimen.

The as-received and rotary-swaged rod steel were cut into different mechanical and fatigue testing specimens, as shown in Figure 4. To ensure that the testing parts of the specimens were all under the same effective strain and deformation conditions, the diameter of all gauge areas were designed to be Φ6 mm. The fatigue specimens were all machined in the same batch to reduce possible errors by turning.

Tensile and compression tests were carried out on an INSTRON-5982 universal testing machine (Instron, Norwood, MA, USA). A WNJ-1000 torsion testing machine (Shanghai Hualong Test Instruments Corporation, Shanghai, China) was used to carry out torsion tests. The above tests were performed with a strain rate of 0.001 s^−1^.

A QBG-100 fatigue testing machine (Changchun Qianbang Test Equipment Co., Ltd., Changchun, China) was used to perform high-frequency fatigue tests and the testing frequency was about 90 Hz. The fatigue tests were performed with a stress amplitude in the range of 420 MPa to 480 MPa. Stress increment between two adjacent stress amplitude was set as 20 MPa. Repeated tests were carried out to eliminate the effect of result scattering.

The Vickers hardness tests were carried out on the entire cross-section surface of the Φ15 mm rod steel. Hardness was tested every 0.5 mm along the two mutually perpendicular diameter lines to reduce the measurement error. A 0.5 kgf load was used in the tests, and the dwell time was set to 15 s.

### 2.3. FEM Settings

**Figure 5:**
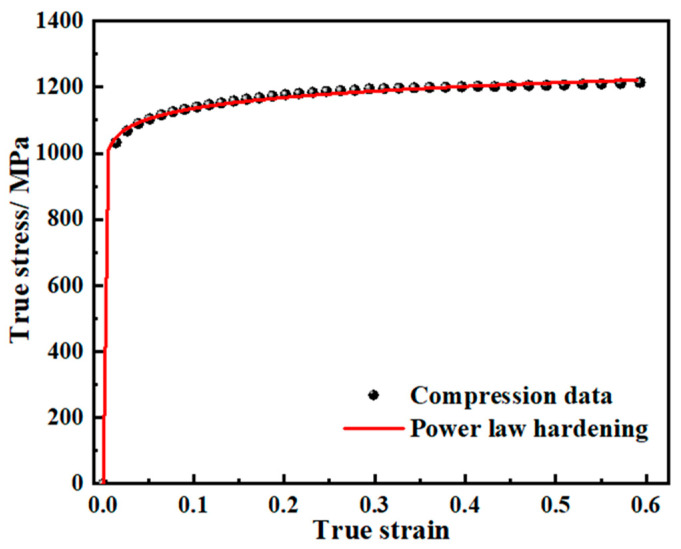
Compression data and power law fitting.

A 3D FE model of the swaging process was constructed according to the actual processing conditions using the commercial finite element code FORGE NxT. The assumption that the hardening of the material was isotropic and uniform was made. Specimens taken from the as-received rod steel with dimensions of Φ6 mm × 9 mm were compressed at a quasi-static rate. The true stress–strain curve obtained from the experiment and fitting data of the power law of hardening ($\sigma =A\varepsilon^{n}$, where *A* = 1247.94 and *n* = 0.041) are shown in Figure 5. The effects of strain rate and temperature were neglected in the cold RS process.

Swaging dies with a circular arc surface were modelled according to the real dies. The circle shape of the closing dies consisted of four eccentric arcs, so the dies could swage the rod to a series of outer diameters from Φ25 mm to Φ15 mm.

RS of the received rod steel is a continuous incremental process, so only a part (with an original deformation length more than 120 mm) of the rod was modeled and simulated as a representative of the whole. To obtain a more precise strain distribution, a dynamic remeshing area was set on the part to be deformed. More than 70,000 elements were meshed in total.

## 3. Results

### 3.1. Microstructure Evolution

**Figure 6:**
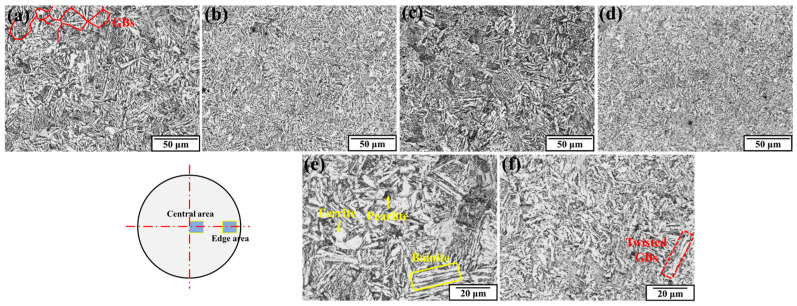
Optical microstructure: (**a**) Central area of as-received rod; (**b**) central area of rotary-swaged rod; (**c**) edge area of as-received rod; (**d**) edge area of rotary-swaged rod; (**e**) magnification observation of as-received rod; (**f**) magnification observation of rotary-swaged rod.

The comparison of the optical microstructure between the as-received steel and the rotary-swaged steel is shown in Figure 6. The observation positions were chosen close to the center and edge of the rod cross-section. The original microstructure mainly consisted of bainite, ferrite, and pearlite. The grain boundaries (GBs) can be observed as the orientation of the changed straight bainite. After the RS process, the bainite and GBs were all severely distorted and bended, and the original straight orientation cannot be observed in any area. This caused the GBs to be difficult to visualize. The RS process caused the severe deformation of the grains along the radial direction, and the GBs were flattened and fragmented.

### 3.2. Tensile Properties

**Figure 7:**
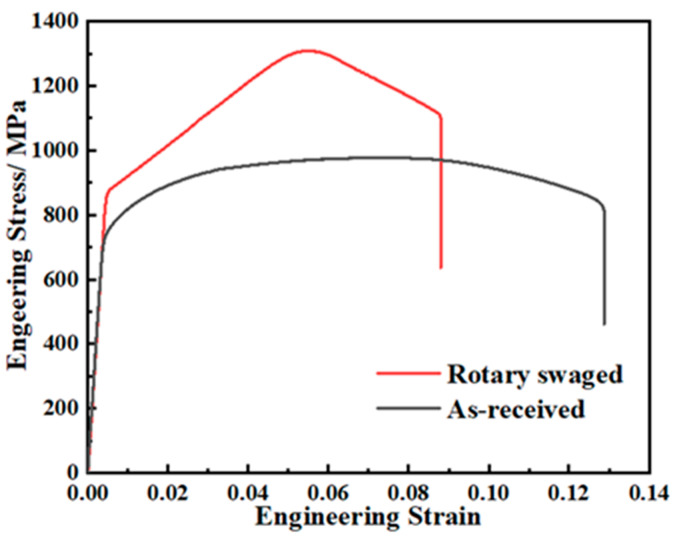
Tensile stress–strain curves.

Figure 7 shows the tensile curves of the as-received and rotary-swaged samples. The tensile property shows a significant difference between them. After RS, the hardening rate of the material was enhanced significantly. The stress increased fairly rapidly to a peak stress, and after the peak stress, the specimen necked and fractured quickly.

The yield strength increased from 749 MPa to 873 MPa, by 16.6%, and the ultimate tensile strength increased from about 977 MPa to 1295 MPa, by 32.5%. The material was significantly strengthened in the process. The elongation strain decreased from 0.127 to 0.087, by 31.5%. The increase in the ultimate tensile strength and the decrease in the elongation are basically in the same proportion.

### 3.3. Torsion Properties

**Figure 8:**
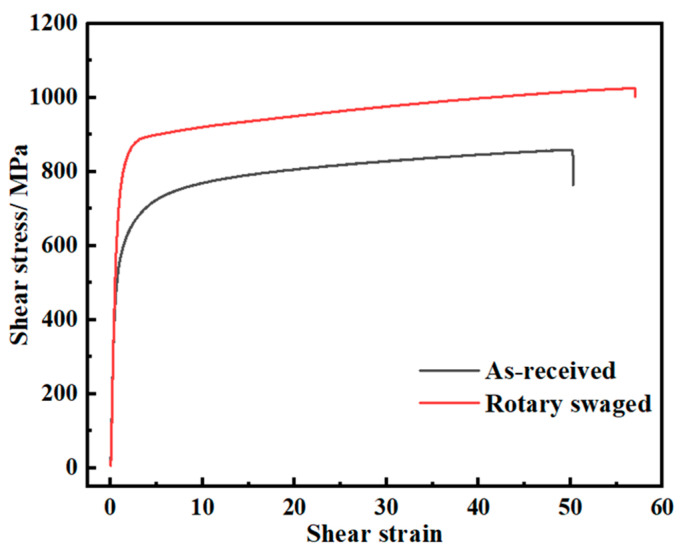
Torsion stress–strain curves.

For the 42CrMo steel used in axle parts, the torsion properties are quite important because the real axle parts bear a large torque under pure torsion conditions in transmission systems and power systems. Figure 8 shows the torsion curves of the as-received and rotary-swaged samples. The torsion yield strength increased from 525 MPa to 752 MPa, by 43.2%, and the ultimate torsion strength increased from 859 MPa to 1025 MPa, by 19.3%. The hardening stage of the material under two conditions was both smooth and flat. Moreover, the maximum torsion angle did not decrease after swaging unlike that of the original material. The clearly different hardening mold between the tension and torsion will be explained in Section 4.

### 3.4. Compression Properties

**Figure 9:**
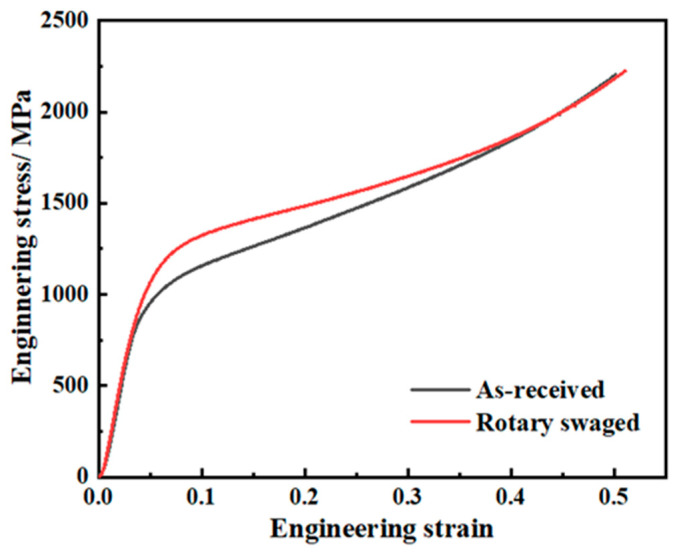
Compression stress–strain curves.

The result of the uniaxial compression test is shown in Figure 9. The compression yield strength increased from 841 MPa to 898 MPa, by 6.8%. Compression softening occurred during the final compression stage of the swaged specimen, especially when the strain reached 0.25. Experiments were carried out several times and no buckling or slippage occurred during compression.

### 3.5. Hardness

**Figure 10:**
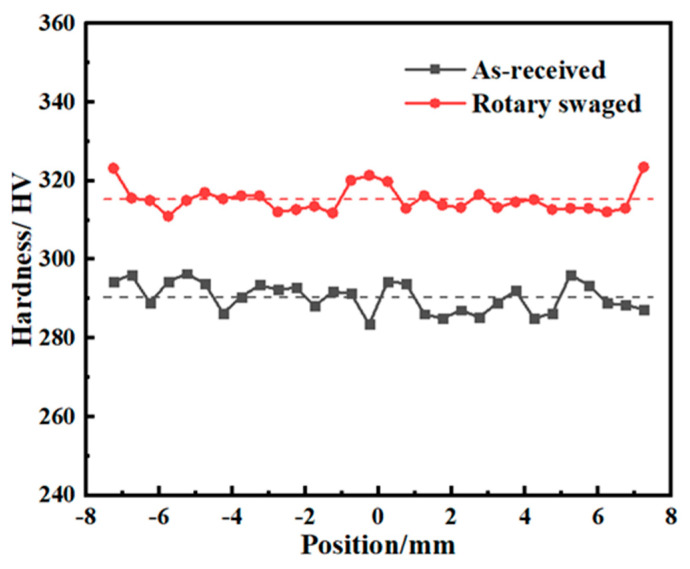
Hardness distributions.

The Vickers hardness across the steel rod section surface is shown in Figure 10. The hardness of both the original and swaged rods was quite uniform, although it showed larger fluctuations in the original rod. After swaging, the hardness at the central area and the near-surface area was relatively higher, and could reach HV323. The average hardness which is signed by the dashed lines increased from HV290.41 to HV315.3, by 8.57%. Although the hardness of the swaged rod in the central area and the edge area showed a relatively higher value, its variation could not be deemed to have led to a different distribution order to the original rod. A hardness distribution with a higher value in the central area can also be seen in AZ31 Mg alloy, 316 L steel, and CoCrFeMnNi high-entropy alloy [16,33,34], and is the most common kind of distribution, as Mao et al. has summarized [35]. However, the hardness of the near-surface area was much higher for the swaged 42CrMo in this case.

### 3.6. Fatigue Properties

**Figure 11:**
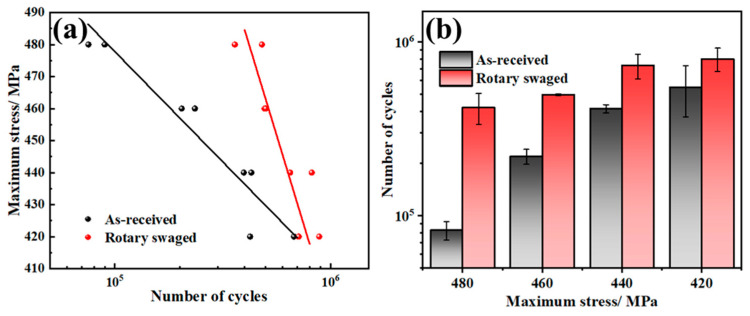
Fatigue results: (**a**) experimental results; (**b**) average life cycles.

The fatigue results are presented in Figure 11. The exponential equation of the *S-NS*–*N* curve is $e^{mS}\cdot N=C$, and it can be derived into another form as S=A+B·lgN. This form was used to describe the relationship between the stress amplitude and fatigue life, and the fitting results give the following equations:

S=821.4−68.73·lgN (As received)

S=1535.73−188.09·lgN (Rotary swaged)

From the average life cycles at two conditions in Figure 11b, the average fatigue life increased by 409.4% at the highest maximum stress (480 MPa) and by 45.2% at the lowest maximum stress (420 MPa).

### 3.7. Tension and Torsion Fracture Observation

**Figure 12:**
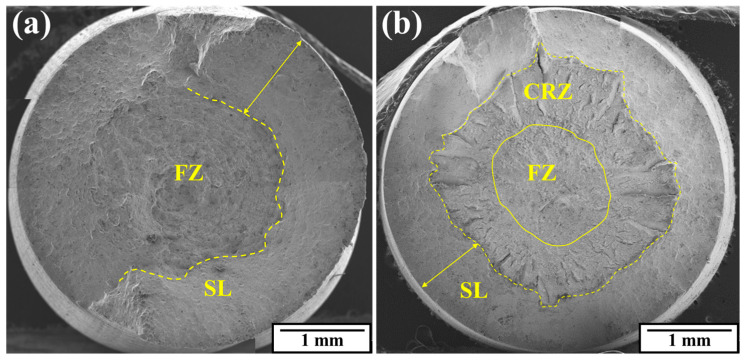
Tensile fracture surfaces: (**a**) as-received; (**b**) rotary-swaged.

Figure 12 shows the surfaces of the tensile fractured specimens under two conditions. After swaging, the fracture mode turned into a standard cup-and-cone fracture morphology, with three distinctly different zones: the fibrous zone (FZ), the crack radial zone (CRZ), and the shear lip (SL). On the as-received surface, the fracture presented a significant failure slope as a whole, while a clear SL was still visible at the same time. In the center of the as-received surface, no common CRZ of the cup cone fracture was found, and the surface also showed a greater roughness, which means that the dimples nucleated and expanded uniformly from different places in the FZ.

Comparing the surfaces under two conditions, we can find that the maximum width of the shear lip decreased from 1.29 mm to 1.00 mm, by 22.5%. The average diameter of the FZ was defined as two mutual vertical measurement values, and it decreased from Φ2.57 mm to Φ1.64 mm, by 36.2%. Based on these changes, it is evident that the composition of the ductile fracture of the swaged rod showed a tendency to decrease, which is consistent with the difference in the toughness and brittleness in the tensile results.

**Figure 13:**
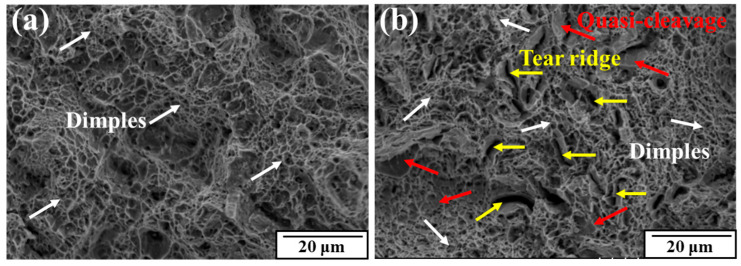
Fracture features on the tensile surfaces: (**a**) as-received; (**b**) rotary-swaged.

In order to form effective contrast, the magnified fracture was observed in the FZ area near the center. The more detailed observation in Figure 13 shows that after RS, a large number of scattered tear ridge features appeared on the fracture surface, which were not found in the as-received state. Also, some quasi-cleavage planes could be observed at different places, indicating that the swaged material was exactly at a transitional state between ductile fracture and brittle fracture. Meanwhile, dimples were still widely present across the surface, so the fracture mode after swaging was still mainly controlled by ductility.

In polycrystalline deformation, grain boundaries play a role in coordinating the deformation of adjacent grains. However, when the grain boundaries are damaged and their deformation ability is weakened, which is not enough to coordinate the deformation of the adjacent grains, the grain boundaries will have a more obvious tendency to become cracked. In the swaged state, edges similar to grain boundaries could be found near the tear ridges, because the dislocations piled up at the grain boundaries, which increased the stress concentration.

**Figure 14:**
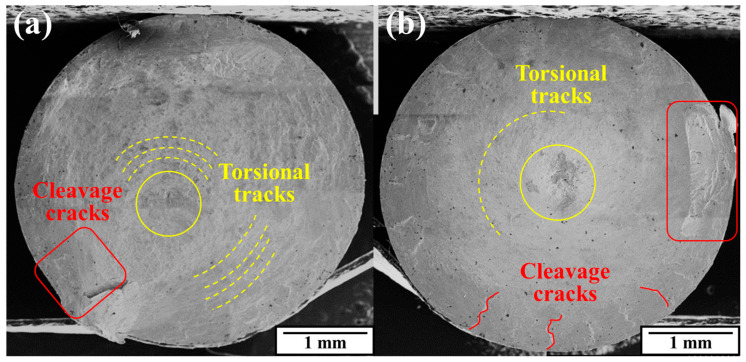
Torsion fracture surfaces: (**a**) as-received; (**b**) rotary-swaged.

Figure 14 shows the surfaces of the fractured torsion specimens under two conditions. The fracture surfaces were both smooth and perpendicular to the specimen axis, indicating that the torsional plasticity was good under both conditions. There was a fracture bulge on the near-surface edge, where the material was rapidly torn under the shear stress when the specimen was finally twisted off. The torsional fracture was much rougher at the original state, which means more uneven shear deformation and plastic strain energy consumption. Also, torsional tracks of plastic expansion could be found and cleavage cracks which are signed by red color were mainly concentrated on the area near the fracture bulge. After swaging, the cleavage cracks could be observed evenly in different places right under the outer surface and all pointed to the center of the specimen along the radial direction. The presumed center area, which was inferred by the distribution of the torsional tracks and shown in the yellow circle, had evidently shifted to near the exact center of the fracture surface. It is interesting to consider that the circumferential performance of the material after RS was more homogeneous; thus, a large number of cleavage cracks uniformly extended after nucleation. Although the final failure still produced a fracture bulge, on which there were cleavage river patterns, the bulge was still much flatter and did not show a shear bevel.

**Figure 15:**
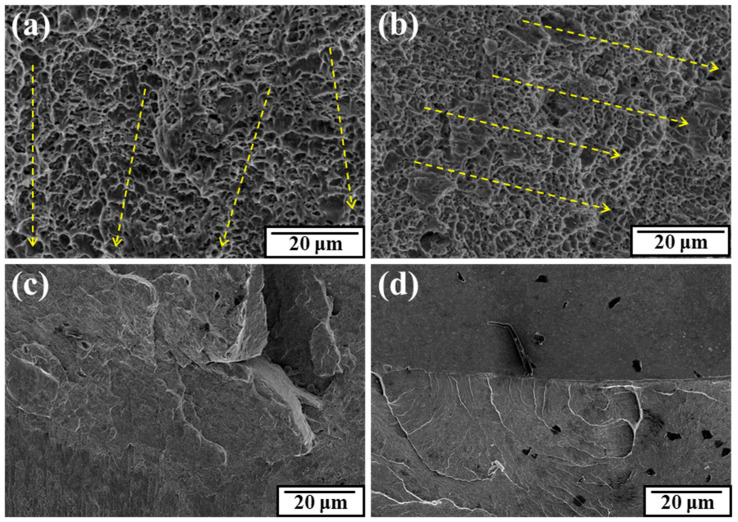
Fracture features on the torsional surfaces: (**a**,**c**) as-received; (**b**,**d**) rotary-swaged.

The magnified fracture was observed in the area that was about 0.5 mm from the center. A more detailed microscopic observation is given in Figure 15. On the areas selected near the center regions, it can be demonstrated that the fracture morphology was dominated by elongated dimples in both samples, which proves that the ductile fracture mode played a dominant role during the fracture process under two material conditions. Due to the presence of torsional shear stress, the dimples on the fracture surface were elongated. For the as-received material, the tracks of torsion deformation (signed by the dashed yellow arrows) did not remain in the same direction, while they were relatively neatly arranged in one direction after RS. Dimples with a radius size less than 1.5 μm were present in a much larger quantity in the rotary-swaged surface. As is universally acknowledged, the main factors affecting the size of the dimples are the size and density of the second phase and the plastic deformation capability. The torsional deformation capability was enhanced after swaging, so the smaller dimples arose because the micropores and second phases had been swaged to a more condensed state in the area with the same dimensions. In addition, fracture bulges on the fracture surface were observed. Cleavage cracks extended outwards from the junction of the bulge. The fairly clear river patterns initiated and converged after swaging, and the regional transition as well as the crack propagation was quite smooth. But for the as-received material, large and coarse cleavage surfaces could be seen and deep cracks had been produced at the transitional region. This also manifested that the torsional plasticity became more uniform after RS.

### 3.8. Fatigue Fracture Observation

**Figure 16:**
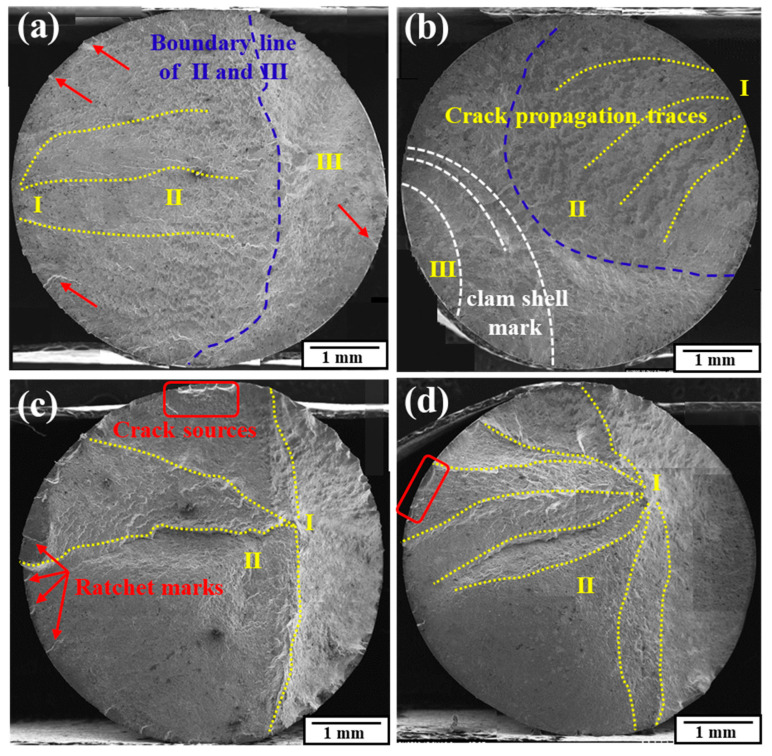
Fatigue fracture surfaces: 440 MPa in (**a**), 480 MPa in (**b**) for as-received specimens; 440 MPa in (**c**), 480 MPa in (**d**) for rotary-swaged specimens.

The fracture surfaces at different stresses are shown in Figure 16. The dashed lines on the surfaces mark the different regions of the fatigue fracture while the dotted lines mark the paths of the cracks. At all stress levels, there were a certain number of cracks near the outer surface for both the as-received and RS specimens.

For the as-received samples, with the increase in the stress level, the fatigue life was shortened and the trend of the multi-sources of cracks increased. The single source of the fatigue initiation could be easily found at 440 MPa but was not that clear when the stress level became higher. Generally, the fracture surface could be divided into three typical zones, which were crack initiation zone I, crack propagation zone II, and fast fracture zone III. But when the stress exceeded 440 MPa, zone II narrowed and disappeared while zone III expanded. It could be found that on the outer edge were distributed a few short cracks, which were oriented towards the specimen’s center; at 440 MPa of stress, lots of short cracks originated in the outer edge; however, when the stress reached 480 MPa, the short cracks could no longer be distinctly observed, while clam shell marks (shown by the white dashed lines) could be found, which implied another patent crack origin on the other side. The clam shell marks usually appeared when the load fluctuated. The average fatigue life at 480 MPa was 82,550 cycles, so it took a short time to finish the experiments. It was reasonable to consider that at such a high stress level, the local deformation exhibited greater inhomogeneity, thus producing local stress fluctuations.

For RS samples, it was quite evident that the fracture surfaces were much rougher. Another significant difference was that there were also evident crack sources in the internal region of the specimen. This phenomenon was similar to the ‘local fatigue’ behavior reported in research on shot peening or deep rolling fatigue [36], which meant that the outer part of the specimen exhibited a clearly higher strength than the inner part, or that the outer part had been severely strengthened. For the rotary-swaged specimens in our study, although a lot of the outer material had been turned, the remaining material still exhibited this phenomenon because of the layered strain strengthening. Apart from the internal crack sources, more crack sources originated from the outer edge, and as the stress level exceeded 440 MPa, long tear ridges appeared in the center part. As the stress increased, the invasion depth of the surface crack source decreased from about 0.49 mm to 0.16 mm, but the internal tear ridge appeared and lengthened from about 2.20 mm to 2.42 mm. At the highest level of stress, at least eight crack propagation paths, which all originated at the internal crack source, were observed, indicating that with the increase in the stress level, the rotary-swaged fatigue fracture was controlled more by the internal crack source than the surface crack source. The multi-sources of the surface cracks gave rise to visible ratchet marks. On the other hand, the number of surface crack sources receded from about 24 to 13. This change also demonstrated that the fracture pattern developed from ‘outer surface-controlled’ to ‘combined controlled’ and finally to ‘internally controlled’.

In summary, the RS process strengthened the material simultaneously in the circumferential direction, leading to the ‘local fatigue’ phenomenon; thus, the cracks that were more likely to originate at the outer edge were inhibited. High stress would limit the crack initiations that were more concentrated in the internal region. Meanwhile, the outer cracks that were initiated would be more likely to invade deeper inward. This led to a competitive relation between the internal cracks and the outer cracks, as a result of which the fatigue life at high stress increased.

**Figure 17:**
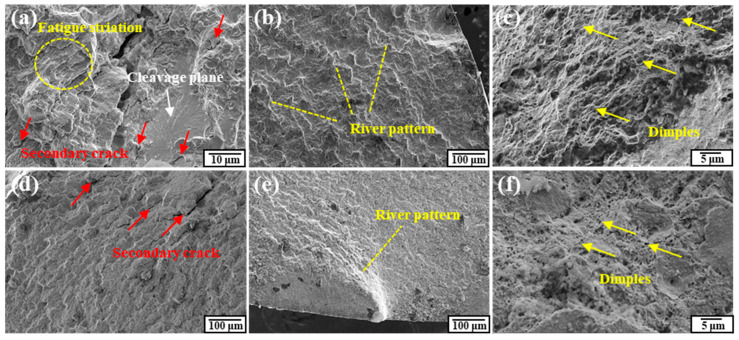
Fatigue fracture surfaces: (**a**–**c**) for as-received specimens; (**d**–**f**) for rotary-swaged specimens.

More detailed micrographs are presented in Figure 17. Cleavage small planes and secondary cracks could both be observed in the as-received and rotary-swaged specimens. The river pattern propagation paths originated at the outer surface and extended inward, resulting in a lot of small cleavage planes. In addition, the dimples found in the as-received specimens could have a size of about 2.4 μm, while the dimples in the rotary-swaged specimens were just smaller than 1 μm or so.

Researchers have proved that environmental factors can cause grain boundary embrittlement in steel material, which is characterized by intergranular cracks [37,38]. However, no intergranular cracks were found in the tested specimens, so it could be concluded that environmental factors had a negligible effect on these fatigue tests.

## 4. Discussion

**Figure 18:**
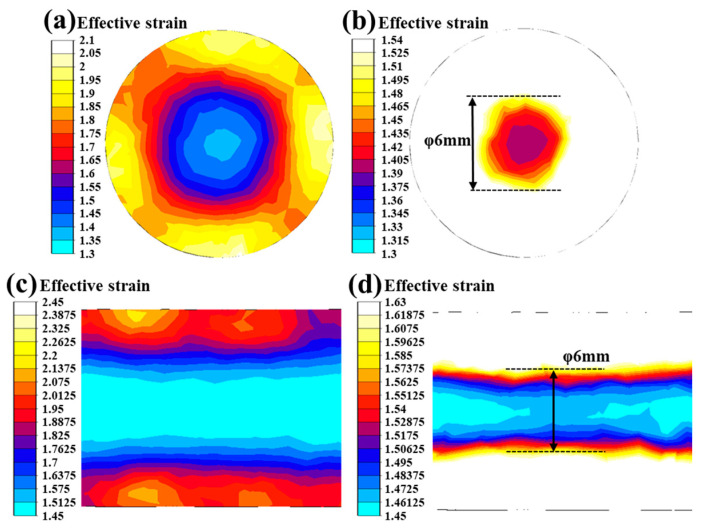
Effective strain after swaging: the whole region in (**a**,**c**); the Φ6 mm region in (**b**,**d**).

**Figure 19:**
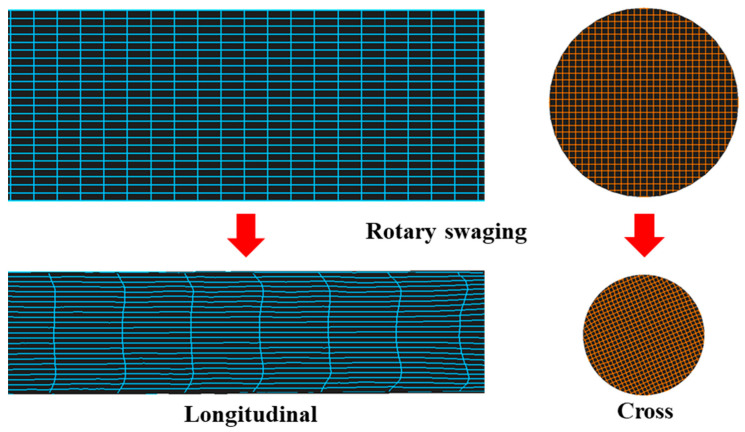
Material orientation change during RS.

The different mechanical behavior of the tension and torsion is analyzed using the FEM results shown in Figure 18 and Figure 19. The effective strain on the longitudinal and cross-sections both exhibited the characteristic of a layered distribution. In the Φ6 mm region, which was equal to the gauge diameter considered, the layered distribution was still very distinct. This manifests as each layer changing with the rod diameter and the effective strain being distributed quite uniformly along the circumferential direction.

After RS with the adopted experimental deformation, the material was clearly elongated along the axial direction. We can demonstrate this through the FEM grid change results in Figure 19. In the longitudinal direction, the material was elongated by around 2.9 times. The material on the surface edge was pulled back and shifted backwards by about 0.9 mm. The influenced depth of the shifted layer was about 1.9 mm and the rest of the inner material could be considered to have been uniformly elongated. On the contrary, the material in the cross-section basically remained uniform during the whole process, though there were some slight grid fluctuations. From the grid result analysis, the conclusion can also be made that the strain remained in a steady distribution on the circumferential direction and it is reasonable to deem the whole rod as multiple more slender rods in the longitudinal direction.

Based on the analysis above, explanations can be further extended:

Bearing a tensile load, the small slender rods of the specimen exhibited different effective strain; thus, the time that they achieved the yield point and failure point was different. When some of the small rods achieved the yield stress, the specimen’s stress–strain curve began to show obvious plasticity, because the yielded small rods would be elongated faster and then they would dominate the specimen’s plastic elongation. But the whole specimen’s stress would continue to rise until enough small rods reached their ultimate tensile strength, because only when adequate material failed would the fracture occur in the macroscopic specimen. This is why the specimen’s ultimate tensile stress increased much more than its yield stress.

**Figure 20:**
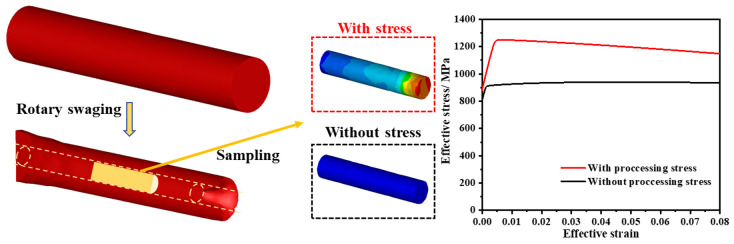
Tension simulation after RS.

In addition, the residual stress produced during RS is believed to be influential. The rod after RS was sampled with the trimming tool in Figure 20, and the stress and strain fields were retained and removed, respectively. Then, the two different samples were simulated under uniaxial tension conditions. The results show that the stress increased sharply and then exhibited a downward trend when the processing stress field was retained; however, the stress remained in a steady state when the processing stress field was removed. The curve trend can explain this problem to some extent, but it should be noted that the curve is not accurate because the trimming process did not release or redistribute the stress field; moreover, the influence of texture and other structural factors were also not included in the simulations.

**Figure 21:**
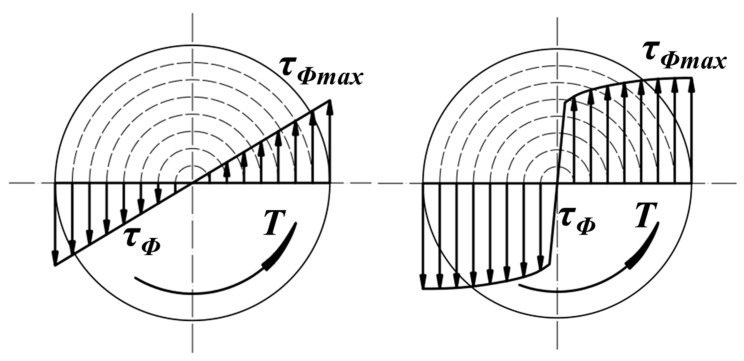
Torque distribution in elastic stage and plastic stage.

Bearing a torsional load, the torque distribution in the elastic stage and plastic stage on the rod’s cross-section would normally be the same as the pattern shown in Figure 21, which consisted of different layers. Along each layer, presented by the dashed circle in Figure 21, the bearing torque remained the same. It was clear that the layered torque distribution pattern coincided well with the effective strain distribution. Materials under the same effective strain could be considered to have been deformed equally; thus, the material in each layer with the same deformation would show very similar hardening behaviors under the same torque. The torque would drive different layers from the outside to the rod center to produce shear slip as the torque increased. Moreover, deformation during RS would make the material more uniform along the circumferential direction but not in the axial direction, so the layered yield and shear slip of the material led to the enhancement in both the torsional yield stress and ultimate torsional stress. The different material uniformity in the bearing directions caused the clearly different hardening mold between the tension and torsion tests.

In addition, it must be mentioned that the residual stress and texture formed in the RS of the bcc metal might also be influencing factors, as discussed in the research of Kocich and Charni et al. [39,40,41]. The mutual effect of all these factors would change the fracture surface after swaging. However, these factors were not considered in this paper.

**Figure 22:**
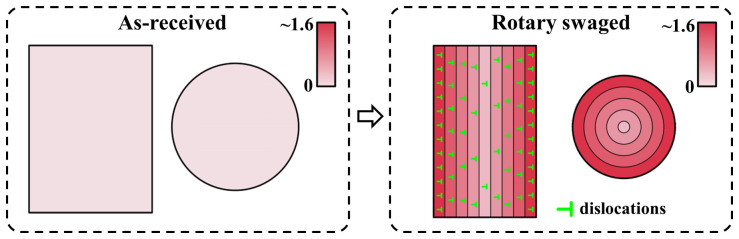
Schematic diagram of compression behavior.

Under the condition of cold RS in steel, the strain is closely related to the dislocation accumulation. Therefore, a possible qualitative explanation for the evolution of the compression behavior is given as follows:

After RS, the hardening degree of the specimen, which is indicated by the gradual red color in Figure 22, increased from the rod center to the relatively outer part. Quantities of dislocations were produced throughout the whole rod and accumulated more at the outer part. This could be induced and verified by the simulations above.

The severe axial elongation caused the accumulated dislocations to arrange along the axial direction. In the uniaxial compression tests, these dislocations were forced to actuate in the reverse direction; thus, the dislocation tangles and obstacles which had been formed during RS were destroyed. In the beginning stage of the uniaxial compression test, the material hardening in the elongation direction played the most significant role, so this resulted in the uniaxial compression property being strengthened, apparently. In the following stage of the test, as more and more tangles and obstacles were destroyed, softening appeared after the strain reached 0.25.

## 5. Conclusions

In this study, the effect of RS on the mechanical behaviors of axle steel rods was investigated. The reason for the differences in the tension and compression properties after RS was analyzed. The factor leading to the improvement in the torsional performance was explained. The evolution process of fatigue life was effectively analyzed. A comprehensive evaluation of the mechanical properties of 42CrMo steel after RS has been conducted, which holds practical significance for the application of RS in axle manufacturing. The main conclusions are summarized as follows:

1. RS greatly enhanced the mechanical properties under torsional conditions, and also improved the ultimate tensile strength significantly. The use of RS technology to manufacture axle parts that mainly bear torsional loads shows promise.
2. The different material uniformity in the bearing directions and residual processing stress field caused the clearly different hardening mode between the tension and torsion tests. The fracture modes showed apparent differences after RS.
3. RS had little effect on the compression properties. Severe axial elongation and uneven dislocations accumulated along the axial direction during RS caused the softening and stress peak to decrease in the compression test.
4. RS could improve the fatigue life of steel, and the competition between internal and external cracks led to the increase in the fatigue life after RS.

## Figures and Tables

**Table 1:** Chemical composition of 42CrMo (wt%).

Element	C	Si	Mn	P	S	V	Cr	Ni	Cu	Mo	Ti	W
wt%	0.40	0.19	0.65	0.015	0.007	0.003	1.002	0.01	0.01	0.17	0.003	0.001

## Data Availability

Data and material will be made available on request.
